# The co-development of personalised 10-year breast cancer risk communications: a ‘think-aloud’ study

**DOI:** 10.1186/s12885-022-10347-3

**Published:** 2022-12-05

**Authors:** Louise S. Gorman, Helen Ruane, Victoria G. Woof, Jake Southworth, Fiona Ulph, D. Gareth Evans, David P. French

**Affiliations:** 1 The Nightingale Centre and Prevent Breast Cancer Centre Research Unit, Manchester University NHS Foundation Trust, Southmoor Road, Manchester, M23 9LT UK; 2 Manchester Centre for Health Psychology, Division of Psychology & Mental Health, School of Health Sciences, The University of Manchester, MAHSC, Oxford Road, Manchester, M13 9PL UK; 3 Department of Genomic Medicine, Division of Evolution and Genomic Science, MAHSC, University of Manchester, Manchester University NHS Foundation Trust, Oxford Road, M13 9WL, Manchester, UK; 4 NIHR Manchester Biomedical Research Centre, Manchester Academic Health Science Centre, Central Manchester University Hospitals NHS Foundation Trust, Manchester, England UK

**Keywords:** Risk communication, Breast cancer, Qualitative, Think aloud, Interviews, Breast screening

## Abstract

**Background:**

Risk stratified breast cancer screening is being considered as a means of improving the balance of benefits and harms of mammography. Stratified screening requires the communication of risk estimates. We aimed to co-develop personalised 10-year breast cancer risk communications for women attending routine mammography.

**Methods:**

We conducted think-aloud interviews on prototype breast cancer risk letters and accompanying information leaflets with women receiving breast screening through the UK National Breast Screening Programme. Risk information was redesigned following feedback from 55 women in three iterations. A deductive thematic analysis of participants’ speech is presented.

**Results:**

Overall, participants appreciated receiving their breast cancer risk. Their comments focused on positive framing and presentation of the risk estimate, a desire for detail on the contribution of individual risk factors to overall risk and effective risk management strategies, and clearly signposted support pathways.

**Conclusion:**

Provision of breast cancer risk information should strive to be personal, understandable and meaningful. Risk information should be continually refined to reflect developments in risk management. Receipt of risk via letter is welcomed but concerns remain around the acceptability of informing women at higher risk in this way, highlighting a need for co-development of risk dissemination and support pathways.

**Supplementary Information:**

The online version contains supplementary material available at 10.1186/s12885-022-10347-3.

## Background

Breast cancer is the most common cancer in women, with invasive breast cancer affecting 55,213 women and causing/leading to 11,399 deaths per year in the UK [1]. To identify breast cancer at an earlier and more treatable stage, 1.87 million women were screened in the NHS Breast Screening Programme (NHS BSP) in 2017-2018 [2].

Risk stratified screening should allow a better balance of harms and benefits [3]. Identifying women at increased risk of breast cancer would allow these women to be offered chemoprevention with tamoxifen or raloxifene, or more frequent screening as per NICE (2013) recommendations [4]. However, many women are not currently identified at increased risk and hence are not offered prevention and early detection options [5, 6]. Notably, there are other potential benefits of providing women with their breast cancer risk such as increasing knowledge and thereby more informed choices regarding screening [7, 8] and prevention options, including potential changes in risk-related behaviours such as reduced alcohol intake [8]. The main potential harms of risk stratified screening include worry and anxiety about cancer in women informed of high-risk [9], and false reassurance in those at below average risk, potentially resulting in subsequent non-attendance at screening [10].

In recent years, a number of web interface tools incorporating multifactorial cancer risk assessment models have been developed, including CanRisk (Breast and Ovarian Analysis of Disease Incidence and Carrier Estimation Algorithm, BOADICEA version 6) [11–13], iPrevent [14] (International Breast Cancer Intervention Study, IBIS, and BOADICEA) and the NCI Breast Cancer Risk Assessment Tool [15]. An additional statistical model for estimating breast cancer risk has been validated for women attending the NHS BSP [16]. This model, based on the Tyrer-Cuzick algorithm (T-C) can provide women with their personalised risk of developing breast cancer, based on information about mammographic density in addition to self-report questions about factors linked to family history of cancer and hormone levels, and, for a proportion, polygenic risk information from single nucleotide polymorphisms (SNPs) [6]. In 2019 the BOADICEA model was updated to incorporate further risk factors and mammographic density [11]. Several studies are underway internationally that offer larger numbers of women personalised breast cancer risk estimates as part of attending routine breast screening [3]. This includes a large UK study to assess the benefits and harms of doing so as a routine part of the NHS BSP [17]. Alongside these studies is an increasing body of work examining best practice in communicating breast cancer risk and risk management options, particularly the Selective Oestrogen Receptor Modulators tamoxifen and raloxifene [18].

Given this progress in risk estimation, it is imperative to develop the best way to communicate such risk to women attending routine breast screening. A review of cancer screening information with guidelines for information in all NHS Cancer Screening Programmes [9] was prompted by a debate about the benefits and costs of cancer screening and how information is communicated [18]. There is evidence that people value receiving written health information and patient information leaflets, however, health information is often of variable quality [19]. Additonally, an estimated 42 % of adults have a low health literacy level [20]. Written information needs to be understood by a diverse range of the general population, it is therefore important to ensure that any new health information is co-produced with the involvement of individuals from the target population. Involving people in healthcare has also been shown to improve satisfaction and quality [21].

There are recognised recommendations on best practice for presenting risk information to the general population, such as the use of absolute risk instead of relative risk, natural frequencies, and giving time frame and frame of reference. In addition, providing a qualitative risk alongside quantitative risk has been shown to facilitate better understanding [22]. However, qualitative and quantitative explanations of risk are often not an effective way to communicate health information on their own but combined with pictures can improve comprehension of health information [23].

In line with this approach, the present research was designed to ensure that communication materials promote good knowledge, to allow informed choices about options, and to avoid harms due to misunderstanding of information presented.

### Objectives of the research

The purpose of this research was to explore how best to inform women attending routine breast screening of their 10-year breast cancer risk. The research aimed to co-produce and refine personalised letters detailing a woman’s risk of developing breast cancer in the next 10 years and an accompanying information leaflet. The leaflet contained standard information on breast cancer risk, signs and symptoms, and signposting for further information.

## Methods

This qualitative think-aloud paper describes a three-step process in the development and refining of breast cancer risk information. The research consists of three steps of development, refinement, and acceptability that were acquired over the course of two studies. The design of the research was informed by four public contributors with experience of receiving personalised breast cancer risk.

### Participants and recruitment

Eligible participants were women aged between 47 and 99 years who attended routine mammographic screening as part of the NHS BSP. All participants were recruited from an earlier programme of work validating the Tyrer-Cuzick risk prediction algorithm (PROCAS) [16]. Participants were randomly selected from the PROCAS database of women who had previously received a letter detailing their personal risk estimate along with an accompanying information leaflet prior to invitation to this research. Women who had previous or current breast cancer were not eligible as this research was concerned with developing information about a woman’s risk of developing breast cancer for the first time and not the likelihood of recurrence.

### Procedure

*Step 1 – appraisal and co-production of letters:* 18 women were presented with initial drafts risk letters and accompanying information leaflets. These were developed based on Cancer Research UK breast cancer leaflets [24] and guidance from the Campaign for Plain English was reviewed, such as using a sans-serif font (Arial) in font size 12. Letters and leaflets were produced for moderate, average and below average-risk women, as women identified as high-risk received a telephone consultation with a clinician. In line with recommendations on developing communications about cancer screening programmes [9], the information aimed to provide accurate information about risk [25] and promote informed choices about screening.

Participants were asked to independently read the version in line with their own risk and to ‘think aloud’ while doing so, verbalising their thoughts to indicate where there are difficulties in comprehension or misunderstandings [26]. If participants were silent for more than 10 seconds, we reminded them to “keep talking”. At the end of this process each woman was then briefly interviewed to elicit further thoughts, questions or comments about the information they had received. The information was then revised following feedback.

*Step 2 – co-production and acceptability:* 19 women were presented with the revised letters and leaflets. These participants followed the same think-aloud process described above to assess changes to risk letters and leaflets in terms of whether they were acceptable, and understandable, and to elicit if any further changes were required.

*Step 3 – acceptability:* 18 women were presented with revised letters and leaflets from Step 2. The revision included new detail on individual risk factors. Step 3 also included a high-risk letter. The letters and leaflets were further developed and personalised through the same co-production process described above.

See Additional file 1: Appendix for example risk letters.

### Analysis

Think aloud interviews were audio recorded and transcribed verbatim. NVivo version 11 was used for data management and coding. Initial analysis focused on coding data using content analysis to make the requisite changes to the documents, so that the working prototype could be embedded into the research programme with immediate effect.

Data were then analysed deductively using thematic analysis from a realist ontological stance [27], centring the analysis on how risk should be presented to enhance understanding and acceptability. Each transcript was systematically read multiple times for familiarisation prior to coding. Coding was carried out at a manifest level allowing for participants’ subjective views to be represented, with the aim of this analysis to inform our understanding of how women comprehend and interpret written risk information. Coding was iterative with emerging codes compared and refined across transcripts. Patterns were identified within the codes and initial themes created. Thematic analysis was conducted by LG, HR, & VW. Codes, emerging themes and the final thematic structure was reviewed and refined by five members of the research team.

## Results

Steps one and two did not include women at high-risk as, at the time, this group were informed of their risk by either face-to-face or telephone consultation. Participants in Step one were aged between 54 and 69 years and in Step two were aged between 54 and 78 years (Table 1). Participants in Step three were younger as this sample (Study two) consisted only of women invited for their first mammogram (Table 2). Participant data extracts have been allocated an identifier which details risk type (for Study one), risk level and participant number e.g. MDA4 = MD [mammographic density] A [average risk] 4 [participant 4], or SM7 = S [SNPs] M [moderate risk] 7 [participant 7]. For Study two, all participant risk was based on Tyrer-Cuzick score and mammographic density, as such data extract are identified by risk level and participant number e.g. A3 = A [average risk] 3 [participant 3].

**Table 1:** Steps one and two participant characteristics

Step 1			Step 2		
10-year breast cancer risk *(based on TC & MD* *or TC, MD & SNPs*^a^*)*	***N*** = 18	Median age (years) at time of interview	10-year breast cancer risk *(based on TC & MD* *or TC, MD & SNPs*^a^*)*	***N*** = 19	Median age (years) at time of interview
Moderate: 5-7.99%	6	66	Moderate: 5-7.99%	8	61.5
Average: 2-4.99%	6	59	Average: 2-4.99%	5	69
Below average/low: < 2%	6	57	Below average/low: < 2%	6	63.5

^a^*TC* Tyrer-Cuzick risk score*, SNPs* Single-nucleotide polymorphisms, *MD* Mammographic density

**Table 2:** Step three participant characteristics

Step 3		
10-year breast cancer risk *(based on TC & MD)*	*N* = 18	Mean age (years) at time of interview
High: ≥8%	5	54
Moderate: 5-7.99%	4	54
Average: 2-4.99%	5	51
Below average/low: < 2%	4	51

### Steps 1&2

#### A need for personalised risk information

Participants stated that there should be two important functions of the letter and leaflet: first to aid women in understanding their risk of developing breast cancer in the next 10 years, and second to understand what they can do about that risk.

Overall, participants were in favour of receiving written risk information through the postal mail. They were appreciative of the opportunity to receive their 10-year risk of developing breast cancer, but highlighted a need for greater personalisation of the risk letters. Unanimously, women wanted to be informed of all of the drivers of their risk (irrespective of their actual risk).

*“Would it be possible to explain why that has been arrived at? Why that level of risk has been arrived at? Because I don’t know whether the fact that I am average-risk is a function of my age or my weight or my medical history, so I think that would be useful to know. I would think that was quite important because if it was due to genetic factors I think that would you mean that you might be more careful, more observant.” (MDA4).*

Participants wanted to know how much each factor contributed to their personal risk of developing breast cancer and whether these are modifiable enough to change risk category. In Study one this was not possible as risk was calculated manually on a person-to-person basis, however, individual risk factors were able to be incorporated into the letters in Step 3.

*“But how much of a weighting is family history and height and weight? So if you said about your weight and height is it going to reduce by how much? And you don’t mention alcohol there do you? I imagine that’s quite a high factor.” (MDA2).*

Participants required detail on effective risk reduction strategies and to be sign-posted to services. In the first modification of the letters, we included new information about reduction of total body weight (if overweight) and its potential impact on breast cancer risk and other diseases.

#### Presentation of risk

Many participants were concerned with the phrasing of risk in the first draft letter. They disliked the explanation of their risk category in terms of how many women will develop breast cancer, and found it anxiety inducing. Instead, they suggested a positive reframing of risk, stating how many women will not develop breast cancer and reported this would be a more *“reassuring”* (SM1) way to communicate risk to recipients.

*“So I think percentages all the way along because that puts it into a bit more perspective even though you are higher risk, your chances of not getting it are 74-92, that’s quite a powerful statement isn’t it?” (SM7).*

All participants discussed ways to present the risk estimate so that women could find that information more easily amongst the main text of the letter. Five women (three average risk and two moderate risk) suggested adding in a diagram that represents their risk in relation to other risk categories.

#### Minimising anxiety

Some participants receiving risk information incorporating both mammographic density and polygenic risk (SNPs) felt that the first risk letter used language that was too “*medical*” (SM3) and too “*complex*” (SL2), thus potentially creating anxiety. This led to a concern that other women would question why they were not tested for high-risk dominant gene mutations such as *BRCA1/2*. To avoid this confusion, those participants recommended removing references to genetic variant tests.

*“I‘d leave that sentence out if it was up to me and I’m not sure about single-nucleotide polymorphisms. Because I think saying you looked for the single nucleotide, but you didn’t test for high-risk genes, if you’re not really into science and even knowledge of breast cancer you’re not going to understand that the high-risk genes are the risk genes that people who have it through their family. And it might just be a bit sort of “well, why didn’t you test me for that. I might have that” (SL2).*

For some women at average-risk, there was a sense of frustration that there is no specific information or pathway for them on how to reduce their risk other than by making lifestyle changes, while simultaneously they lacked the reassurance of being at below average risk.

*“This paragraph here and when I was I reading this again today I said to my husband, and I said “hmmm it’s just average” and he said “well that’s good isn’t it” but I don’t think it was probably set out clearly enough in this risk factor. Um and then this here ‘there’s things that all women can do’ … and I thought that was just a little bit of an ‘ok you’re average’, that’s it basically, go away and watch your diet.”(*SA6*).*

### Step 3

#### Identifying with breast cancer

Participants offered suggestions on the aesthetics of the risk letter and leaflet. They were keen to have information that was overtly identifiable with breast cancer causes and suggested a redesign of the accompanying leaflet cover to reflect this. Many participants wanted a softer colour scheme and more pictures, while still maintaining a dominance of pink as a colour most identifiable with breast cancer.

*“I would have just assumed that if it was something to do with breast cancer there might be some breast pictures or a picture of a lady or something, rather than a window.” (*A4*).*

#### Comprehension of the written information

When assessing the content of the risk information, participants liked the positive framing of risk; however, five participants shared a preference for the risk to be presented in percentages. In the Step 2 letter, risk was presented as a narrative expression of percentage, for example by stating that “5 to 7 out of one hundred women in your risk category” instead of “5-7% of women in your risk category” and some women found this confusing. There was also a need to reiterate that the risk provided was a 10-year risk estimate and not lifetime risk, in order to make risk more meaningful and also to minimise misinterpretation.

*“It’s a numbers thing. It’s how you read the number. So, when I looked at it, five to seven, and I thought, out of ten … No, it’s out of 100. It’s how you read that number … And, again, you know, here we’ve got the same headings, but I would put the percentages there. And then the timeframe here, within ten years of the mammogram, so, presumably, what we’re saying is, we don’t know, or we’re not making any comment about your chances of developing breast cancer more than ten years out.” (M2).*

For some participants, presenting risk estimates in percentages was considered more acceptable than a narrative presentation of risk, increasing perceived validity of the risk estimate.

*“They’re using the actual numbers rather than percentages. It feels really personal, you know, about people. Percentages might be a little bit softer, I don’t know, or more scientific.” (L4).*

### Risk categories may induce anxiety

Participants in the second Study were concerned about the naming of the risk categories, in particular the high-risk and the moderate-risk groups. Concerns stemmed from the potential to create anxiety if the risk category was misinterpreted. These participants would prefer not to use the term ‘high-risk’, however only one participant gave a suggestion as to how this group should be termed.

*“But being told I’m high-risk and I’m possibly going to get it … that’s it, because you don’t know when you’re going to get it. Maybe not even say ‘high-risk of getting it’: that you have been identified as an at-risk category.” (H1).*

Explaining the preference for change, participants explained that focus should be on the consequences of being moderate or high-risk. These participants desired more explanation of what that risk means in terms of likelihood of developing breast cancer.

*“Moderate, I think. Yes, moderate risk and … I think there would be a nicer way to put it, but without … it’s hard to think of it but, you know. Like just sort of, whilst this … this assessment, you know, shows that your risk was calculated to be moderate, then this isn’t a guarantee. It tends to mean that five and seven out of a hundred, so they were really close together, from a mammogram with this score, will move on to get breast cancer.” (*A5*).*

### Signposting to discuss risk management

In response to concerns about the letter potentially raising anxiety, it was suggested that a hotline should be provided so that women would have a point of contact in-between receiving the letter and attending a risk consultation.

*“I just wonder whether there could be, you know, a number that they could ring and speak to somebody sooner rather than later. I’m just kind of thinking, because you might get a bit anxious while you’re sort of waiting for your appointment.” (*H3*).*

Participants required an explanation of what a Family History Clinic is; where their local clinic is based; and how one should be referred to discuss risk. Many participants at average or below average-risk felt that the names of the breast cancer prevention drugs (tamoxifen and raloxifene) in the information leaflet could be omitted. These participants felt that this information was only relevant to women at higher risk and could lead to individuals researching the drugs online, increasing the potential for misinformation. Some participants felt that such detail is best left to shared decision-making during a risk consultation.

*“It might cause a kneejerk reaction to a lot of people to go, right, I want that drug. And then it’s … so I think moneywise, it’s more money that the NHS are spending which they haven’t got the funds to spend and but I do like that but I don’t think that should be put in the letter. That should possibly … if someone wants to discuss the risk and they go to a face-to-face or telephone appointment, consultation, then that should be discussed then.” (A3).*

## Discussion

This qualitative research found that women attending breast screening were positive about receiving written breast cancer risk assessment information through the postal mail. Participants stated a preference for the information to be visually aligned to breast cancer causes and not to be overly medical in its description of genetic risk factors or mammographic density. They wanted detail about how each risk factor contributed to their overall risk of developing breast cancer in the next 10 years. They were concerned about the naming of risk categories, particularly for the high-risk group often assuming risk is much higher than it is and that a graphical presentation of risk is preferred over narrative presentations. Women desired to be told of effective risk reduction strategies, and to be provided with a means of directly contacting relevant healthcare professionals should they have questions about their risk estimate. Although the work here focuses on the communication of 10-year breast cancer risk estimates, many of the results are also applicable to the communication of lifetime risk.

A key finding in the present research was the overall acceptability of the approach proposed: receiving personalised risk information was welcomed. Tailoring health information has long been considered an effective method of improving behavioural outcomes, including cancer prevention and detection recommendations, by increasing perceived relevance [28]. Our research found a need for continuing refinement of personalisation in breast cancer risk communication, the reported benefits of which lie in understanding and having faith in one’s personal risk estimate in order to reduce anxiety and to consider appropriate risk management pathways. Adopting a form of identifiable health branding by embedding the letters in pink colours, symbolic of breast cancer causes was desirable. Health branding has been shown to develop and reinforce relationships and encourages exchange [29].

Participants were concerned about the presentation of risk in the letters. They proposed that a graphical presentation of risk and risk to be stated in percentages would be beneficial, rather than as a narrative. This was seen to reiterate the timespan that the risk estimation is valid (in this model, to reiterate that risk was presented in 10-year estimates and not lifetime risk). This is in line with previous literature supporting the use of numerical presentations of risk [30], and using graphical representations facilitate understanding of risk information [31]. The finding that presenting risk as an event rate, in our example as number of women likely to not develop breast cancer out of 100, is felt to be better understood than merely presenting percentages [31], is somewhat at odds with evidence on risk presentation. The literature generally suggests that the use of natural frequencies, i.e. the number of events and the number of people in the population results in better understanding [32].

Participants were keen to know whether risk factors were modifiable, however whether this is for information only or relates to behavioural intentions is not clear in our data. Previous research suggests that few people are aware of the link between lifestyle and cancer risk despite up to 40% of cancers being attributable to lifestyle factors [33–35]. Our participants wanted detail on effective risk reduction strategies without too much emphasis on behaviour for risk management, which could suggest that the message of modifiable cancer risk factors is challenged by fatalism or a lack of belief in the effect of lifestyle change on cancer risk. It is certainly the case that provision of personalised risk information does not produce the large or sustained changes in behaviour that are often proposed [36, 37].

Relatedly, our results show a participant preference for positively framed risk, so that the expected number of women who do not develop breast cancer in each category is given. Much of the previous literature on message framing focuses on a comparison of gain or loss framing impact on behavioural change [38, 39], however our key focus was on reduction of anxiety rather than impacting on screening behaviour.

Since this work was conducted, the letters and leaflets have been given to a separate group of women receiving risk estimates for the first time [40]. This produced encouraging results, as the women who received these materials reported lower levels of anxiety than a comparison group of women who did not receive risk estimates, largely due to reductions in anxiety in women at lower risk [40]. Further, satisfaction with information was good, and an assessment of understanding also showed generally good levels of understanding [40]. There was some evidence that women given risk estimates that were partly based on SNPs felt they understood the information they were given less well, in line with the findings reported here. Overall, these findings support the idea that the present research achieved many of the objectives set out here.

### Limitations

Although we approached women who had previously received their personal breast cancer risk as part of a large epidemiological cohort study, our sample lacked ethnic and educational level diversity, with the sample consisting of only three non-White women and all participants having at least secondary education. This may reflect greater issues in uptake to breast screening among non-white women, combined with the bias of a self-selected sample who had chosen to participate in research and opted to receive their 10-year breast cancer risk estimate. In addition, research participants had previously opted to receive their personalised breast cancer risk so were not viewing the risk letters for the first time as a clinical cohort would be, therefore the impact of emotional response on understanding breast cancer risk information was not explored. We recommend that researchers and breast services seek to explore the development of breast cancer risk communication information and materials with women from a range of ethnicities and educational level [41, 42]. Future research is including the contribution of personal risk factors in personalised letters, and will examine the impact of this [17].

### Practice implications

Implementing personalised breast cancer risk effectively into the NHS BSP means identifying effective yet practical strategies for communicating risk that take into account the large volume of individuals accessing cancer screening. Our participants considered it acceptable to receive risk information via postal mail, but were concerned about whether this is appropriate for women at high-risk, highlighting a need for the co-production of cancer risk dissemination pathways. Provision of a hotline to facilitate communication and answer queries may enable women to feel supported in receiving their breast cancer risk by postal mail and could reduce anxiety. Telephone support has been suggested for individuals accessing other screening programmes [43]. Future research should explore help-seeking and the impact of support provision in this context.

## Conclusion

Effective communication of breast cancer risk needs to be, personal, understandable, and meaningful for individuals. Providing personal 10-year breast cancer risk to women attending routine breast screening is acceptable, however further co-development of risk letters is needed as breast cancer risk models continue to be expanded to include multiple risk factors. Additional provisions should be made to support women experiencing anxiety.

## Supplementary Information

**Additional file 1.**

## Data Availability

This publication is supported by multiple datasets. Due to the sensitive nature of the research, interviewees consented to their data being retained or shared on an individual basis. Anonymised interview transcripts from participants who consented to data sharing may be available upon reasonable request from the corresponding author.
