# Supplementary material for: The co-development of personalised 10-year breast cancer risk communications: a ‘think-aloud’ study
Source: BMC Cancer. 2022 Dec 5;22:1264. doi: 10.1186/s12885-022-10347-3 (PMC9721070; doi:10.1186/s12885-022-10347-3)
Supplement: Supplementary file 1 — Additional file 1. [file 12885_2022_10347_MOESM1_ESM.docx]

**APPENDICES: Example risk letters by step of development**

**Example risk letter (Step1):**

Dear **[INSERT NAME]**

When you attended a screening mammogram on **[INSERT DATE]**, you agreed to take part in the PROCAS study. At the time you joined the study, you also indicated on your consent form that you would like to be informed of your risk of breast cancer.

The questionnaire you completed asked you to give us some background information about yourself such as:

- Your current weight and height
- Family history of breast cancer
- When you started your periods
- If you are taking HRT or contraception
- How much exercise you currently do

Your risk is made up from a combination of these factors along with your breast density (as assessed from your mammogram). We have calculated your risk of developing breast cancer in the next 10 years form the information you provided us with on the questionnaire and the mammogram you had at the time you joined the study.

*(Only in risk letters incorporating polygenic risk):* *As part of the PROCAS study, you also provided us with a saliva (spit) sample. In addition to the risk factors outlined above, we know that your risk can be affected by small changes in your DNA called single-nucleotide polymorphisms (or SNPs). SNPs are small changes to your DNA that might increase or decrease your risk of developing breast cancer*

Your risk of developing breast cancer in the next 10 years was calculated to be **high/moderate/average/below average (delete as appropriate).** This means that between 5 and 8 women out of 100 with risk factors like you will develop breast cancer within 10 years of that mammogram.

There are things that all women can do to reduce their risk of breast cancer, such as maintaining a healthy body weight through diet and exercise. More information on the signs and symptoms of breast cancer and ways to reduce your risk are given in the accompanying leaflet.

Please note: these results are based on the information you gave when you joined the study. Risk may change over time, however as this is a research study and risk assessment is not yet part of routine clinical care, unfortunately we are unable to re-calculate risk for you.

If you have concerns that your risk may have increased significantly and you think you may be eligible for referral to a family history clinic, please see your GP.

Yours sincerely,

**Example risk letter (Step 2)**

Dear **[INSERT NAME]**,

**RE: PROCAS study – Predicting Risk of Cancer at Screening**

**NHS number:**

Thank-you for taking part in the PROCAS study. As part of that study, when you attended your mammogram on **[INSERT DATE]**, we asked you to complete a questionnaire where you gave us some background information. Your risk is calculated from a combination of factors which are outlined in the accompanying leaflet. We have calculated your risk of developing breast cancer in the next 10 years from the information you have provided us with on the questionnaire and your breast density (the amount of tissue in your breast that is not fat) as assessed from your mammogram.

Your risk of developing breast cancer in the next 10 years was calculated to be **above average (moderate) risk.** This means that out of 100 women with risk factors like you, between 5 and 7 women will develop breast cancer **within 10 years of that mammogram.**

**High**

8 to 26 women out of 100 will develop breast cancer.

**74 – 92 of those women will NOT develop the disease.**

**Above average (moderate)**

5 to 7 women out of 100 will develop breast cancer.

**93-95 of those women will NOT develop the disease.**

**Average**

2 to 4 women out of 100 will develop breast cancer.

**96-98 of those women will NOT develop the disease.**

**Below average**

0 to 2 women out of 100 will develop breast cancer.

**98 of those women will NOT develop the disease.**

**Within 10 years**:

There are things that all women can do to reduce their risk of breast cancer, such as losing weight (if needed) through diet and exercise. Being overweight increases the risk of developing breast cancer. If you are overweight, losing at least 5% of your weight and keeping it off can reduce your risk of breast cancer by around 25%, even if breast cancer is in your family. Losing weight can also help reduce your risk of developing other diseases, such as heart disease, diabetes and dementia. More information on the ways to reduce your risk together with signs and symptoms of breast cancer are given in the accompanying leaflet.

**If you want to discuss you risk further, please make an appointment with the study team on [study team telephone number] who will arrange either a telephone consultation or face-to-face appointment at your local Family History Clinic.** During this appointment, your breast cancer risk will be explained to you along with information on the ways in which to reduce your risk that you may be eligible for, such as taking a drug (tamoxifen or raloxifene) for prevention.

In summary, you have an increased risk of developing breast cancer compared to other women in Manchester. Please remember that 93-95 women out of 100 women in your risk group will **not** develop breast cancer in the next 10 years.

Yours sincerely,

**If you think your risk estimate is not accurate**

The risk estimate is based on the information you gave to us when you joined the study. Risk may change over time, however as this is a research study and risk assessment is not yet part of routine clinical care, unfortunately we are unable to re-calculate your risk for you.

If you have concerns that your risk may have increased significantly and you think you may be eligible for referral to a family history clinic, please see your GP.

**If you have been diagnosed with breast cancer**

The speed at which we receive information about who has been diagnosed with breast cancer depends on which hospital you were diagnosed at. If you have been diagnosed with breast cancer since joining the study, please contact us on [study team phone number] and we will be able to send you a more relevant letter.

**Queries about your breast screening appointments**

If you have a query regarding breast screening, please call [breast screening service telephone number], or email: [breast screening service email address]

**Example risk letter (Step 3)**

**RE: [study name]**

**IMPORTANT: ALL CLEAR after your recent mammogram. This is NOT a recall.**

Thank-you for taking part in the [study name] study. As part of that study we asked you to complete a questionnaire before you attended your mammogram. In this questionnaire you gave us information which enabled us to work out your risk of developing breast cancer in the next 10 years. Your risk is calculated from a combination of factors associated with your family history, lifestyle and breast density (the amount of tissue in your breast that is not fat).

Your risk of developing breast cancer in the next 10 years was calculated to be **above average (moderate) risk.** This means that 5 - 7% of women in your risk category will develop breast cancer **within the next 10 years.**

The table below provides you with information about your risk category and where you are in relation to other women:

**High**

**80 to 92% of women will NOT develop breast cancer.**

8 to 20% of these women will develop the disease.

**Above average (moderate)**

**93 to 95% of women will NOT develop breast cancer.**

5 to 7% of these women will develop the disease.

**Average**

**96 to 98% of women will NOT develop breast cancer.**

2 to 4% of these women will develop the disease.

**Below average**

**98% of women will NOT develop breast cancer.**

0 to 2% of women will develop the disease.

**YOUR RISK**

Based on your questionnaire answers and mammogram, the following may have increased your risk of breast cancer:

- **[INSERT RISK FACTOR 1]**
- **[INSERT RISK FACTOR 2]**
- **[INSERT RISK FACTOR 3]**

However, these factor(s) may have helped to prevent your risk from being any higher:

- **[INSERT PREVENTATIVE FACTOR 1]**
- **[INSERT PREVENTATIVE FACTOR 2]**
- **[INSERT PREVENTATIVE FACTOR 3]**

Your risk of breast cancer can be reduced by up to 25% by making positive lifestyle changes, such as; adopting a healthy diet, taking regular exercise and losing weight (if needed). Such changes can also help reduce your risk of developing other diseases, such as heart disease, diabetes and dementia. More information on the ways to reduce your risk, together with the signs and symptoms of breast cancer are provided in the accompanying leaflet.

**If you would like to discuss your risk further, please make an appointment with the study team (Telephone: study team phone number) who will arrange either a telephone consultation or face-to-face appointment at your local Family History Clinic*, [INSERT HOSPITAL].** During this appointment, your breast cancer risk will be explained in more detail and further information will be provided on how to reduce your risk. For example, you may be eligible to take preventative medication.

Please remember that even though you have an increased risk of developing breast cancer in the next 10 years, **93 to 95% of women in your risk group will NOT develop the disease.**

Should you have any questions please get in touch with the BC-Predict Study team on **[study team telephone number]**, Monday to Friday 10am to 2pm.

Yours sincerely,

*A Family History Clinic (FHC) is a centre where healthcare professionals advice and counsel individuals who have been identified as having an increased risk of developing breast cancer.

**Queries about your breast screening appointments**

If you have any questions about your routine breast screening appointments, please call **[INSERT LOCAL SCREENING OFFICE NUMBER]**, or email: **[LOCAL SCREENING OFFICE EMAIL].**
